# High-throughput sequencing of *Astrammina rara*: Sampling the giant genome of a giant foraminiferan protist

**DOI:** 10.1186/1471-2164-12-169

**Published:** 2011-03-31

**Authors:** Andrea Habura, Yubo Hou, Andrew A Reilly, Samuel S Bowser

**Affiliations:** 1 Division of Infectious Disease, Wadsworth Center, New York State Department of Health, PO Box 509, Albany, NY 12201, USA; 2 Division of Translational Medicine, Wadsworth Center, New York State Department of Health, PO Box 509, Albany, NY 12201, USA; 3 Division of Laboratory Operations, Wadsworth Center, New York State Department of Health, PO Box 509, Albany, NY 12201, USA; 4 Department of Biomedical Sciences, School of Public Health, The University at Albany, Empire State Plaza, Albany, NY 12201, USA

## Abstract

**Background:**

Foraminiferan protists, which are significant players in most marine ecosystems, are also genetic innovators, harboring unique modifications to proteins that make up the basic eukaryotic cell machinery. Despite their ecological and evolutionary importance, foraminiferan genomes are poorly understood due to the extreme sequence divergence of many genes and the difficulty of obtaining pure samples: exogenous DNA from ingested food or ecto/endo symbionts often vastly exceed the amount of "native" DNA, and foraminiferans cannot be cultured axenically. Few foraminiferal genes have been sequenced from genomic material, although partial sequences of coding regions have been determined by EST studies and mass spectroscopy. The lack of genomic data has impeded evolutionary and cell-biology studies and has also hindered our ability to test ecological hypotheses using genetic tools.

**Results:**

454 sequence analysis was performed on a library derived from whole genome amplification of microdissected nuclei of the Antarctic foraminiferan *Astrammina rara*. Xenogenomic sequence, which was shown not to be of eukaryotic origin, represented only 12% of the sample. The first foraminiferal examples of important classes of genes, such as tRNA genes, are reported, and we present evidence that sequences of mitochondrial origin have been translocated to the nucleus. The recovery of a 3' UTR and downstream sequence from an actin gene suggests that foraminiferal mRNA processing may have some unusual features. Finally, the presence of a co-purified bacterial genome in the library also permitted the first calculation of the size of a foraminiferal genome by molecular methods, and statistical analysis of sequence from different genomic sources indicates that low-complexity tracts of the genome may be endoreplicated in some stages of the foraminiferal life cycle.

**Conclusions:**

These data provide the first window into genomic organization and genetic control in these organisms, and also complement and expands upon information about foraminiferal genes based on EST projects. The genomic data obtained are informative for environmental and cell-biological studies, and will also be useful for efforts to understand relationships between foraminiferans and other protists.

## Background

The Foraminifera are an abundant and widespread group of marine protists, noted for their ability to construct fossilizable shells or "tests". Some foraminiferans, such as members of the planktonic genus *Globigerina*, are so abundant that their empty tests dominate the biotic material in about 35% of the Earth's seafloor [1]; half of the CaCO_3 _deposited in the deep ocean is in the form of foraminiferal tests [2]. The early-evolving groups within the Foraminifera possess either nonmineralized organic tests, or agglutinated tests built from materials gathered from the environment [3]. Because foraminiferans are large and predatory protists, they are also prominent members of meio- and macrofaunal food webs and have been so throughout the Phanerozoic [4]. The cytoskeletal modifications necessitated by their large size and dynamic microtubule-driven reticulopodia have also made them favored targets for cell-biology investigations [5-7].

Despite the geochemical and biological importance of foraminiferans, they are poorly understood genetically. Because these organisms usually have translucent or opaque shells and are often multinucleate, chromosome numbers have been estimated for only a few species. Previous authors have reported between 7 and 24 chromosomes [8].

Only partial SSU rDNA sequence is available for the great majority of the ~250 species analyzed to date, and only a few genes are available for the remainder (reviewed in [9]). This dearth of data is primarily due to difficulties associated with conventional methods for gene discovery, such as amplification with universal primers and generation of cDNA libraries. These techniques often fail in foraminiferans, due to sequence divergence in many foraminiferal genes, the difficulty of obtaining adequate amounts of pure RNA or DNA, and the fact that actively growing foraminiferans often harbor eukaryotic endosymbionts such as dinoflagellates. Indeed, the first sequence data reported from a foraminiferan, obtained using universal SSU rDNA primers [10], was later discovered to have been from an alveolate endosymbiont. The much more divergent foraminiferal SSU rDNA was only identified later, after primers specific for the group were developed [11].

The foraminiferan for which the largest amount of genomic data is available is the unusual "naked" foraminiferan *Reticulomyxa filosa *[12]; an EST analysis of this species [13] identified 1059 nonredundant sequences. The sample is probably not exhaustive, and was also derived from a species which cannot be cultured in the absence of other eukaryotes, making additional genomic information highly useful. A similar but somewhat smaller EST dataset, comprising 672 sequences from the multilocular foraminiferan *Quinqueloculina*, is also available [14]. Due to their nature, neither dataset contains information on non-coding regions of the foraminiferal genome.

**Figure 1:**
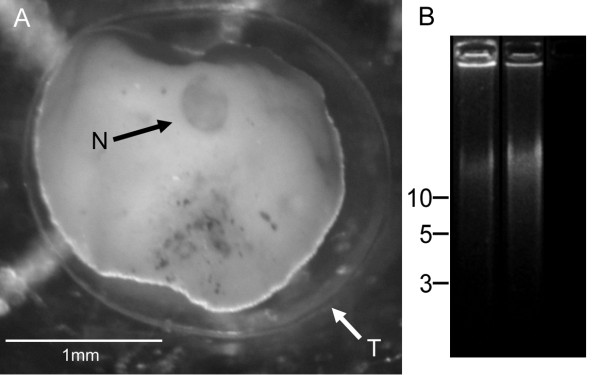
**Source material**. A. Features of *Astrammina rara *morphology. Individual organisms consist of a large cell with a durable theca (T), encased in a loosely bound agglutinated outer covering that can be removed (as in this image) without injuring the cell. Vegetative individuals usually have a single nucleus (N) that is often 0.5 mm in diameter or more, although multinucleate individuals, with approximately 20-30 smaller nuclei, are also observed. B. Products of WGA on an *A. rara *nuclear template. Left: *A. rara *DNA, 5 nuclei. Center: Positive control (purified DNA from the foraminiferan *Allogromia laticollaris *and associated organisms). Right: Negative control.

The giant Antarctic species *Astrammina rara *possesses several unusual properties that make it a good subject for foraminiferal genomic studies. Cold-water agglutinated foraminiferans are less likely to harbor contaminating endosymbionts than are their temperate or tropical relatives, so this species is more likely to yield DNA that is uncontaminated by xenogenomic material of eukaryotic origin. While most mature foraminiferans are multinucleate, *Astrammina *cells generally contain a single nucleus that can be >500 μm in diameter (see Figure 1). The nucleus has an unusually thick and impermeable nuclear membrane and can be isolated mechanically from the rest of the cell without rupturing. A single nucleus contains ~ 2 ng of DNA (Habura, C. Hayden and Bowser, unpublished observations), but this figure most likely represents hundreds or thousands of genome copies because foraminiferal genomes are thought to be endoreplicated at certain stages of the life cycle [15,16]. Extracted nuclei from *Astrammina rara *cells are therefore an unusually good source for pure foraminiferal genomic DNA, although the quantity obtained is necessarily limited.

Genomic DNA extracts can be amplified for sequencing using multiple-displacement amplification (MDA; [17]). MDA is considered to be the most unbiased of genome amplification techniques [18,19], and is especially useful for genomic analysis of small, pure samples from unculturable organisms [20]. In the present study, we used MDA to amplify genomic DNA obtained from isolated *Astrammina *nuclei, and the amplified DNA was then sequenced using 454 technology. We report the first substantial body of non-coding genomic data from a foraminiferan, provide an estimate of genome size in these organisms, and use statistical methods to reveal information about genome structure. Our approach overcomes many of the refractory problems in foraminiferal genomics and provides vital data for further studies of the foraminiferal genome.

## Results

### Nuclear isolation and DNA purification

The predicted genomic composition of isolated *Astrammina rara *nuclei was assessed by examination of two nuclei stained with SYBR Green I (Habura and A. Khodjakov, unpublished observations). The outer surface of the nuclear membrane was coated with a thin layer of material, presumably cytoplasmic in origin, which showed punctate staining on a scale consistent with mitochondria adhering to the nuclear surface. The interior of the nucleus stained less intensely with SYBR Green I. No other masses were observed. The stained nuclei were not used as sources of DNA for the study, due to the risk of contamination with exogenous DNA, but we consider them to be representative of the isolated nuclei.

DNA purified from 5 isolated nuclei was used as a template for a 50-μl whole-genome amplification reaction (Repli-g, QIAGEN) according to the manufacturer's instructions, and the product of the MDA reaction was analyzed by gel electrophoresis. The reaction yielded 37 μg of large (>40 kb) DNA amplimers, indicating that the template was undegraded and had been efficiently amplified (Figure 1B).

Foraminiferal isolates carry the risk of exogenomic contamination. To test whether the eukaryotic DNA in the sample was truly derived from the foraminiferan, we challenged the whole-genome amplified template with both foram-specific and universal eukaryotic primers for Domain III of the SSU rDNA (see [11]). The sequences from both reactions were excellent matches (>99% identity) for previously reported sequences from *A. rara*. While the universal primer sr10r is not a perfect match for the *A. rara *SSU rDNA, due to the extreme sequence divergence of foraminiferal SSU's, sequencing of the 5' and 3' ends of the products of the "universal" amplimers indicates that they are the product of mispriming on an *A. rara *template. Because non-foraminiferal eukaryotes' SSU rDNA would have been more strongly favored as a template had they been present (in that the non-foraminiferal SSU rDNA is a better sequence match for the primers), we concluded that the sample is not detectably contaminated with the DNA of other eukaryotes.

### Library creation, 454 sequencing, and contig assembly

Sequencing of the library created from the whole-genome amplified sample resulted in 234,301 successful reads, representing 49,036,585 bases with a mean of 209 bases per read. At the time of sequencing, the GS FLX platform could achieve 250 bp per read under optimal conditions, which indicates that the library was of good quality and was being sequenced successfully. The reads were assembled into contigs using Newbler assembly software, which is integrated into the GS FLX system. Reads were assembled into 3314 total contigs, 875 of which were "large" (i.e., >500 bp in length); 51% of the reads were deemed to be from repeat regions.

Surprisingly, 96% of the reads overlapped, with 60% of the positions having at least 2X coverage. This degree of coverage for the entire genome is highly unlikely: some genes that had previously been sequenced from *A. rara *were not identified in the protein-coding genes identified in the contig population (see discussion below), and only a few hundred genic contigs were identified in any event. We therefore conducted an analysis of the contig population to determine the genomic origin and depth of sampling represented.

### Initial sequence analysis of contig population

BLAST searches (*blastn *and *tblastx*) were performed on the entire dataset to identify the probable origin of each contig. Although the foraminiferal genomic DNA had been prepared by meticulous methods designed to eliminate as much exogenous DNA as possible, 12% of the contigs could be identified by BLAST search as being of non-eukaryotic origin, in that they showed similarity e <1 × 10^-5 ^to bacterial or viral sequences and had no eukaryotic matches of that quality. 149 contigs (1725 reads, 74,225 bases) had their closest matches in the genome sequence of the psychrophilic bacterium *Colwellia psychrerythraea *[GenBank:NC003910]. These reads are very likely to have derived from the genome of the Antarctic bacterium *Colwellia rossi*, which is known to be present at the harvesting site. An additional 219 contigs (5064 reads, 119,838 bases), which were generally shorter and lower-confidence than the presumptive *Colwellia *reads, were identified as deriving from bacteria other than *Colwellia*. Some matches were to close relatives of *Colwellia*, especially other Alteromonodales (γ-proteobacteria) such as *Shewanella *and *Pseudoalteromonas*. Therefore, it is possible that some or all of these reads are also derived from *C. rossi. *55 contigs (22,483 reads, 73,508 bases) most closely matched bacteriophage, eukaryotic viruses, or virus-like sequences such as transposable elements. These reads may derive from organisms infecting the foraminiferan, from bacteriophage infecting the co-purified bacteria, or from transposable elements within the foraminiferal genome.

The remaining 2891 contigs either contained sequence that was clearly of eukaryotic origin (match of < e ^-5 ^to known eukaryotic sequence), or had no strong matches to any sequences in GenBank. Of these, 148 had at least 30% of the positions occupied by N's. These contigs were deemed unanalyzable and were removed from the dataset; they are not considered further here. The remaining contigs were considered to be of eukaryotic origin even if no sequence match was found, on the reasoning that eukaryotic genomes usually contain long tracts of low-complexity sequence for which no reliable matches can be identified. The contigs generated from this study have been deposited in GenBank as [GenBank:ADNL01000001-ADNL01003231].

The overall GC content of the reads is 42%. However, the content of specific subpopulations of the reads varies. Sequences identified as bacterial but not *Colwellia *have higher GC contents than average, at 45%. Sequences specifically identified as deriving from the *Colwellia rossi *genome are 39% GC, a figure entirely consistent with the GC content of *C. rossi's *sister species C. *psychrerythraea*, which is 37.9% [21]. Contigs showing similarity to viruses and transposons are 40% GC.

### Comparison to other foraminiferal genome-scale datasets

The newly generated eukaryotic sequences were compared to the two pre-existing large foraminiferal datasets, from the genera *Reticulomyxa *and *Quinqueloculina*. 10 of the *Astrammina rara *contigs have *tblastx *matches e <1 × 10^-5 ^in the *Reticulomyxa *dataset, which are identified as containing sequence from actin 1 and 2, alpha- and beta-tubulin, and polyubiquitin. A *tblastx *search of the two EST datasets against each other showed that 64 of the 672 *Quinqueloculina *sequences have matches e <1 × 10^-5 ^to sequences in the *Reticulomyxa *dataset. These matches consist of sequences derived from the same genes identified in the *Astrammina-Reticulomyxa* comparison, as well as alpha-tubulin 3 and elongation factor 1A. In general, the best BLAST match for a randomly chosen sequence in one of these datasets is not found in the other datasets.

### Features of genic eukaryotic contigs

One hundred and thirteen of the eukaryotic contigs contained high-confidence gene sequence, defined as a match with an e-value of <1 × 10^-5 ^against a known eukaryotic sequence (see Additional File 1). Approximately 200 more sequences showed weaker similarities to eukaryotic proteins, although in some cases these may simply represent repetitive tracts of rare amino acids found in unrelated peptides. Many of the strongest matches were to sequences from foraminiferans or other members of the Rhizaria. Contig 1403 contains an SSU rDNA sequence which was clearly derived from *Astrammina rara *(e <1 × 10^-50^), confirming that *Astrammina *genomic DNA had been successfully recovered during library construction. Two contigs, 2915 and 14, showed strong sequence similarity to actin genes described from foraminiferans and from *Gromia*, another protist thought to be closely related to foraminiferans [22]. Contig 32 contained sequence similar to a ubiquitin/ribosomal protein s27a fusion reported from *Bigelowiella*, another rhizarian protist [23].

Other contigs contained the first foraminiferal examples of several other functional classes of genes. Contig 3051 comprises a cluster of 7 eukaryotic tRNA genes: Gln, Ala, Leu, Ser, Lys, Thr (AGT), and Thr (CGT). Several other contigs also contained tRNA genes. None of the genes were present in arrays, as is the case for *Entamoeba*. Contig 3052 contained an aldolase sequence, Contig 1839 was an excellent match for histone 2, and sequences from ribosomal proteins were identified in five contigs.

In addition, conserved functional domains shared by many eukaryotic proteins were also identified in the dataset. Twelve contigs encoded apparent DEAH-box or DEAD-box domains, suggesting that they contain coding sequence for proteins with a role in RNA processing. Five encoded predicted ankyrin repeats. Contig 56 contained 6 coding regions that correspond to the transmembrane domains of G-protein coupled 7-transmembrane receptors; it may contain sequence from a divergent member of this gene family. Contig 3296 clearly contains sequence from an ABC transporter protein gene.

**Figure 2:**
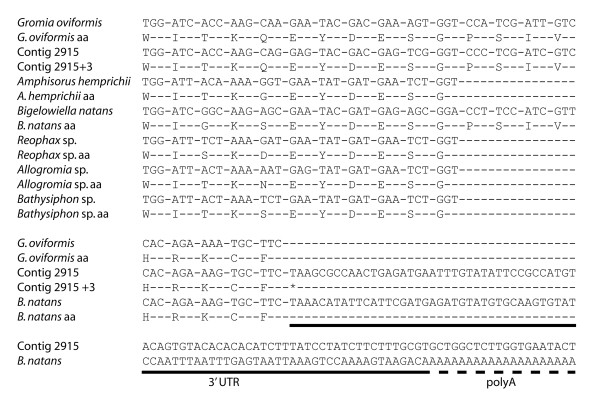
**Identification of a coding region boundary**. Contig 2915 contains coding sequence with strong similarity to foraminiferal and rhizarian actin genes. The 5' end of the contig contains predicted coding region for the C-terminus of an actin gene, as well as sequence which is expected, based on comparison to mRNA data obtained from the rhizarian *Bigelowiella natans*, to be the 3' UTR. The 3' end of the contig, which spans an additional 1.3 kb, contains no identificable genic sequence.

Genic contigs also provide important information about gene structure in foraminiferans that was not previously available from EST projects. Contig 2915 contained the first coding region boundary recovered from a foraminiferan (see Figure 2). The first 719 bp of the contig (with the exception of a Type II intron comprising nt 202-393) are alignable with the 3' ends of several reported rhizarian actin genes. One of these previously reported sequences, AY251793 (from *Bigelowiella natans*), was derived from mRNA and includes 96 bp of 3' UTR and the poly-A tail that marks the end of the transcript. A potential polyadenylation signal, ATTAAA, lies at -18 from the start of the tail. Contig 2915 contained 1350 bp of sequence 3' of the end of the coding region, which showed no homology to the equivalent region in the *Bigelowiella *transcript or to any other sequence in GenBank. No polyadenylation signal was identified in the foraminiferal sequence, although whether this absence was due to the use of non-canonical signals or divergent mechanisms for polyadenylation in foraminiferans is not known.

Some of the identified genes also provide important clues to macromolecular function in foraminiferans. For example, Contig 2707 contained two adjacent exons (separated by a 141-bp Type II intron) of a gene with strong homology to eukaryotic ribosomal protein S18. This protein is located at the top of the "head" of the small subunit of the ribosome, whose RNA core is formed by Domain III of the SSU rDNA [24]. This region of the SSU contains several novel helices and other expansions in foraminiferans [25]; in fact, foraminiferans as a group have the largest SSU rDNA genes of any eukaryotic taxon. Identification of the ribosomal proteins which assemble in this region is an important step toward understanding the contribution of foraminiferal SSU rRNA modifications to the structure of the foraminiferal ribosome.

### Foraminiferal mitochondrial-like genes

Five of the contigs (110, 1256, 2679, 2804, and 2876, comprising 248 reads in total) contained sequences that most strongly match mitochondrial genes derived from protists or other eukaryotes. These contigs contained regions of lower-confidence basecalling, which is potentially a sign of polymorphism. The matches were not extremely robust and showed no clear taxonomic bias, which may be due to the paucity of mitochondrial sequences reported from the Rhizaria. As described above, the genomic origin of these sequences is also unclear; while the nuclear microdissection technique that we describe could have carried some mitochondrial genomes into the primary genomic extract, mitochondrial genes have also been found to have been transferred to the nucleus during the evolution of many eukaryotic lineages [26].

In order to better understand the genomic origin of the reported mitochondrial sequences, we designed primer pairs based on the contig sequences which should allow amplification of diagnostic regions of the mitochondrial genome (see Materials and Methods). The primers were used to challenge a WGA *A. rara *template. Seven distinct amplimers were obtained, sequenced, and identified by BLAST search. These sequences have been deposited in GenBank as [GenBank:HM119593-HM119599].

Interestingly, most of the amplimers represented partial gene fusions or tandem repeats. They were all non-transcribable and are likely to be mitochondrial pseudogenes. The first amplimer (HM119593) comprises a 1236-bp sequence, of which the 5'-terminal 40 bp was identical to the COX1-like contig 110. The following 150 bp also appeared to be COX1-like. The 3'-terminal 235 bp of the amplimer were identical to the COX3-like contig 2876. Three other amplimers of varying sizes also showed homology to COX1 on the 5' end and COX3 on the 3' end, and contain 300-700 bp of intervening sequence with no strong homology to any other known gene.

### Statistical distribution of contigs assigned to different classes of genomic origin

**Figure 3:**
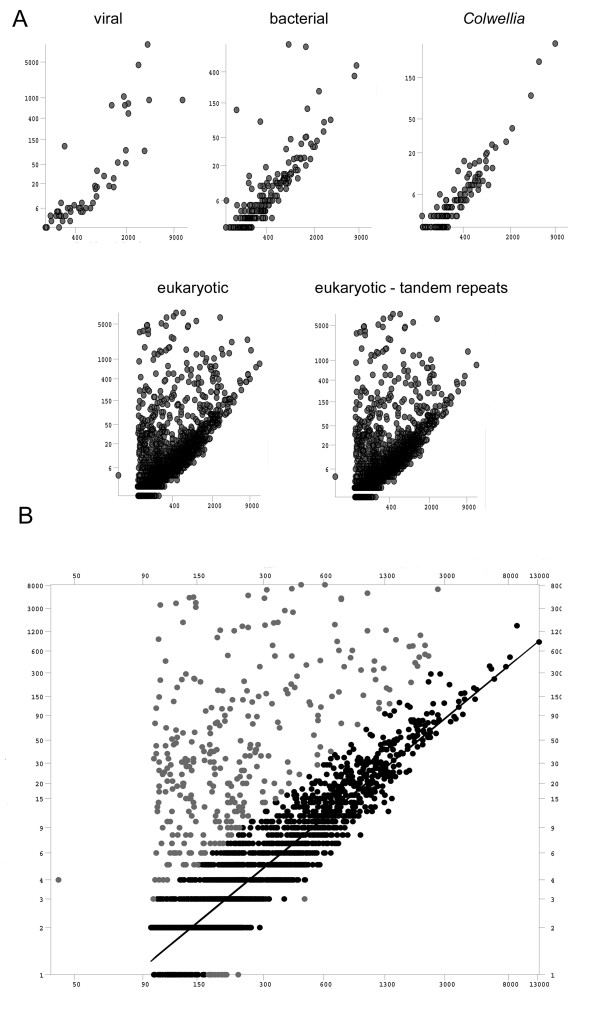
**Statistical analysis of contig populations**. A: Log:log plots of contig length (X axis) vs. number of reads (Y axis) for identified sub-populations of contigs. Viral and bacterial sequences (upper row) show a linear relationship between contig length and number of reads, but eukaryotic contigs (lower row) contain a sub-population of contigs which have an unusually high number of reads for their length. Sequences containing tandem repeats contribute to this phenomenon but do not explain it entirely, as a large proportion of these "overread" contigs remain after removal of tandem repeats (lower right). B: Regression analysis of eukaryotic contigs with tandem repeats removed. Most contigs show a linear relationship between contig length and read number (black), but a second population of contigs does not (gray). This population contains disproportionately few genic contigs. X axis: log-log of length. Y axis: log-log of number of reads.

If the whole-genome amplification and library construction process was unbiased, the number of reads represented in a single contig should have been directly proportional to the length of the contig. This relationship clearly held for the sequences identified as of bacterial or viral origin (see Figure 3A). However, the population of presumably eukaryotic contigs contained a large number of sequences which are "overread" for their length. Such overreading can be caused by the presence of tandem repeats; if several singleton reads derive from a long tandem repeat region, they may be erroneously judged to be overlapping by the assembly software and, as a result, be assembled into a shorter contig that appears to be unusually heavily sampled. Therefore, we used the SERV applet [27] to identify contigs containing tandem repeat regions that spanned more than 100 bp. Because the mean read length for the sequencing reaction was 208 bp, tracts of this size would be sufficient to allow erroneous overlapping assembly and thereby an altered ratio of length to number of reads. The analysis was also run on the presumptive bacterial and viral sequences, which should not contain tandem repeats, as a test of the robustness of the algorithm.

Of the non-eukaryotic sequences, only 7 contigs (1.6%) were identified as containing candidate tandem repeats, and most of these were low-quality: i.e., they were relatively short and consisted of imperfect repeats. In contrast, 184 eukaryotic contigs (6.7%) contained identifiable tandem repeats >100 bp, and many more contained shorter tracts. Several of these tandem repeats represent candidate microsatellite sequences, which will be discussed in a future report. When these sequences were removed from the eukaryotic contig population (Figure 3A, fifth panel), the distribution of length:numread ratios became, surprisingly, more strongly bimodal: the greater part of the population showed a distribution very similar to the linear relationship exhibited by the bacterial and viral sequences, but there appeared to be a residuum with very high numbers of reads per unit length. The marginal distribution of numbers of reads identified two populations of contigs, suggesting that there were in fact two populations present. A robust regression of the number of reads as a function of contig length for this dataset (Figure 3B) with a weight threshold of 0.62 (consistent with a Gaussian distribution governing the model's errors) results in identification of 12% (324/2805) of the points as outliers. Only 4% (12/324) of the longer sequences had unexpectedly few reads for their length, while the remainder exhibited an excess of reads. Within this population, 38% (120/312) exhibited a large excess of reads, from 59 to 8000. 220 contigs had weights below 0.5, and were therefore far from the expected length:numread ratio. Interestingly, a comparison of genic content by weight indicated that the overread contigs were gene-poor; none of the 38 contigs with weights below 1 × 10^-3 ^(representing 56,827 reads, or 24% of the total read population for the run) contained any identifiable gene sequences. The few contigs that were "underread" for their length were generally singleton reads, and probably represent unusually successful individual sequencing reactions.

## Discussion

*Genome coverage and WGA. *60% of all bases in the dataset overlapped at least once, suggesting a very strong level of coverage of the genome. However, comparison with the *Colwellia rossi *genome, combined with the absence of sequence for some known foraminiferal genes, indicates that the coverage was not comprehensive. It is most likely that the Repli-g process amplified a random subset of the genomic material present in the sample, and that these amplimers were then read to approximately 2X coverage (based on the statistical analysis of the contig population) during 454 sequencing. Because all of the genomes present in the sample showed this pattern, it seems likely that the subsampling represents a template-independent artifact introduced by the mechanics of the whole-genome amplification process, rather than unusual genome structure or organization in the foraminiferan. Such artifacts have been reported in other high-throughput sequencing reactions performed on MDA-amplified genomes. For example, a recent study using Illumina sequencing on an MDA-amplified single- cell *Procholorococcus *genome extract [19] reported stochastic subsampling, with 1,000-fold differences in the relative amplification of different portions of the genome. Similar subsampling has also been observed in other studies [28].

### Comparison to other foraminiferal datasets

Surprisingly, there is relatively little overlap between the three genomic-scale foraminiferal datasets. The two EST datasets share less than 10% of their sequences, and these are matches to a small number of genes. Most of these consist of cytoskeletal genes, which is not surprising; these datasets should be dominated by proteins that are heavily transcribed in the living foraminiferan. Foraminiferans have extensive and highly motile cytoskeletons, and microtubule extension and remodeling is essential to the formation of the reticulopodia. Only 3% of the *Astrammina *dataset overlaps the *Reticulomyxa *dataset, but it is of genomic origin, and therefore also comprises genes of lower transcription levels and extensive tracts of non-coding sequence.

While it might be surprising that the datasets do not overlap more strongly than they do, this observation may reflect the small sample sizes and the relatively long evolutionary distances between different foraminiferal species. The LCA of *Astrammina, Quinqueloculina *and *Reticulomyxa *probably dates to approximately 800 million years ago [3], and even well-conserved genes such as the SSU rDNA are partially unalignable between these species [9].

### Presence of introns in foraminiferal genes

Single-celled eukaryotes exhibit considerable variation in the extent to which genes contain Type II (spliceosomal) introns; some protist genomes have relatively few or no Type II introns, and absence of these introns seems to correlate with alteration of the carboxy-terminal domain of RPB1 [29]. Unfortunately, sequence data for the CTD of RPB1 is not available for foraminiferans [30], partially because the 3' end of the gene appears to be divergent and is difficult to amplify. Canonical spliceosomal introns have already been identified in SSU rDNA (Habura and Bowser, unpublished), actin [31], and tubulin [32] genes in foraminiferans, indicating that the suspected alterations in the CTD of RPB1, whatever they may be, do not affect intron splicing. As described above, several contigs in the present study also contained canonical spliceosomal introns, which therefore appear to be a common feature of foraminiferal genes.

### NUMT and foraminiferal mitochondrial DNA

While mitochondrial-like sequences were recovered during the present study, the evidence suggests that none of these contigs represent mitochondrial genomic DNA. We conclude that the *A. rara *genome contains pseudogene sequences of mitochondrial origin, commonly referred to as nuclear mitochondrial DNA (NUMT), as has also been seen in other eukaryotes [26]. No genuinely mitochondrial sequence was recovered during this study. However, the primer sets described here may not have been well designed for foraminiferal mitochondrial genomes. We are currently engaged in purification of DNA from reticulopodia (the foraminiferal "pseudopods"), which contain mitochondria but no nuclear DNA. Material recovered will be used as a template for mitochondrial genome discovery.

### Colwellia rossi and calculated genome size

The presence of *Colwellia rossi *DNA in the sample allowed us to calculate an approximate genome size for *Astrammina rara*. The genome of *C. rossi*'s sister species *C. psychrerythraea *strain 34H [GenBank:NC003910] is 5.4 Mb in size. The length of all identified *C. rossi *contigs in the present dataset is 74,225, which should represent ~1.4% of the *Colwellia *genome. If the other bacterial contigs are also considered to be derived from the *Colwellia *genome, for reasons described above, they contribute an additional 119,838 bp in length for a total of 194,063 bases or 3.5% of the genome. Since neither of the bacterial contig sets shows evidence of statistical overreading, it is probable that these contigs represent an accurate sub-sampling of the bacterial genomes present.

Calculation of the percentage of the *Astrammina *genome sampled is less straightforward. As described above, there are two sub-populations of the eukaryotic contigs, which complicates the assessment of the sampling efficiency. Tandem repeat sequences probably represent a larger tract in the genome than is represented by their aggregate length. In addition, we identified 38 contigs with extremely high numbers of reads, which formed a statistically distinct population and were not genic. It seems most likely that they are derived from low-complexity regions in the genome, but we cannot exclude the possibility that they were the result of a peculiarity of WGA when used on foraminiferal DNA. In either case, it seems most prudent to exclude them from a calculation of genome size that uses the statistically more homogenous bacterial sequences as a comparison group.

The population of eukaryotic contigs that are statistically comparable to the bacterial sequences consists of 2667 contigs with a total length of 1,108,533 bp. If only the bacterial sequences that were confidently identified as being of *Colwellia rossi *origin are used as a comparison group, then the calculated genome size of *Astrammina rara *is ~79 Mb. If all bacterial sequences are presumed to be of *Colwellia *origin, the equivalent figure is ~32 Mb. If all presumed eukaryotic sequences (including tandem repeats and overread sequences) are included, with a total length of 1,294,660 bp, the corresponding calculated genome sizes are 92 Mb and 37 Mb respectively. The possibility that repeat regions were artificially "telescoped" during read assembly probably means that these figures should be considered a lower bound for the genome size of *A. rara*. A calculated genome size of 80-100 Mb is also compatible with an estimate of the haploid genome size of the foraminiferan *Allogromia laticollaris *(83 ± 29 Mb), derived from the intensity of DAPI staining of gamete nuclei [33].

The DNA content of an individual *A. rara *nucleus at this stage of the life cycle is ~2 ng (Habura, C. Hayden and Bowser, unpublished observations), suggesting that the ploidy of the cells used in the present study is on the order of 10,000. A large body of morphological data (for a discussion, see [9]) has demonstrated that foraminiferans undergo considerable endoreplication during some phases of their life cycles, and the vegetative *A. rara *cells used here are in a phase of the life cycle in which genome copy number is predicted to be high. Our results represent the first direct molecular demonstration of the correctness of this interpretation.

This study also sheds light on the genomic changes that may take place during *zerfall *and gametogenesis in foraminiferans and related protists. *Zerfall *is a nuclear reorganization and "cleansing" what takes place just before a series of very rapid mitotic divisions which produce tens to thousands of gametes. If the overread sequences in the current dataset represent endoreplicated regions within the vegetative genome, then *zerfall *could be a process of reorganizing the genome and eliminating overreplicated regions, essentially returning the vegetative genome to "germline" and preparing it for multiple mitotic divisions. If this is true, foraminiferal genomes may be highly dynamic both in copy number and in content throughout the life cycle.

## Conclusions

In summary, our results represent the first sizable body of sequence information about the non-coding regions of the foraminiferal genome. This includes examples of gene clusters, evidence of gene transfer in the form of NUMT, and documentation of extensive endoreplication of parts of the genome during certain phases of the life cycle. We believe that it will help shed light on unusual aspects of genome organization in foraminiferans which have long been mysterious, including the premitotic genome processing known as *zerfall*. We hope that the information obtained will inform future studies of genomic architecture and control in these organisms. In addition, the study introduces novel statistical and methodological approaches that should be of use to other researchers working on challenging microbial taxa.

## Methods

### Specimen collection

The top 1 cm of sediment at a water depth of 20-25 meters was collected by scuba divers in Explorers Cove (S 77 34.552 E163 31.742), an embayment of New Harbor, western McMurdo Sound, Antarctica, using a portable airlift dredge (detailed in [34]). At the surface, the >1 mm residue was transferred to a refrigerated tray and sorted for *Astrammina rara *under a stereomicroscope. Living specimens were placed in 1 L Nalgene jars with ~600 ml of filtered seawater before refrigerated (-2°C) transport to laboratory facilities in Albany.

### Genomic extract preparation

The agglutinated tests of *Astrammina rara*, which are not tightly bound to the cell surface, were individually removed using two needle-nose forceps [35]. After test removal, cells were washed with several changes of sterile artificial seawater (Instant Ocean, Blacksburg, VA) and incubated for 2 days, in order to allow the cells to digest recently consumed prey. Only cells that showed clear signs of viability, particularly the extension of reticulopodia, were used for nuclear isolation. Nuclei were removed from cells with a pair of fine glass needles, retrieved with a Pasteur pipet, and placed in 100 μl TE buffer until DNA isolation. DNA was purified from the nuclei within one hour of microsurgery. All steps were performed in a chiller bath set at -2°C.

### DNA purification and whole-genome amplification

DNA was purified from pooled nuclei from 5 cells using the DNEasy Plant Mini Kit (QIAGEN), eluted with a final volume of 30 μl. 5 μl of this isolate was used as a template for Repli-g (QIAGEN) whole genome amplification (WGA) according to the manufacturer's instructions.

1 μl of the amplification product was run on an 0.8% agarose gel for determination of product quality. 1 μl of a 1:10 dilution of the WGA product was used as a template for nested PCR using the foraminiferan-specific SSU rDNA primers s14F3a/B and s14F1/s20r, and the universal eukaryotic SSU rDNA primers sr10r/B as described previously [25]. Products of amplification were cloned into pGEM-T Easy vector (Promega) and replicated in *Escherichia coli *strain JM109. Individual clones were purified with the SpinPrep mini kit (QIAGEN), and six clones for each product were sequenced using primer M13 on a PE-Biosystems ABI PRISM 377XL automated DNA sequencer.

### Library preparation, sequencing, and contig assembly

The WGA DNA was used to construct a library for 454 sequencing, using the GS FLX Standard DNA Library Preparation Kit (Roche) according to the manufacturer's instructions. The prepared library was sequenced on 1/8 of a PicoTiter plate on the GS FLX system housed in the Mark Welch laboratory at the Marine Biological Laboratory, Woods Hole, MA. Reads were filtered for quality and assembled into contigs using the de novo assembly tool integrated into the GS FLX platform. Statistics for the assembly process are shown in Additional File 2.

### Data analysis

Assembled contigs were assessed to determine the likely genomic source for the sequence data. An initial search using *blastn *was used to identify very strong matches to foraminiferal genes, and to segregate contigs that showed strong matches to previously reported bacterial or viral sequences. The remainder of the dataset was analyzed using *tblastx *and contigs were assigned to eukaryotic, bacterial, or viral/bacteriophage populations based on the analysis. All contigs with fewer than 70% of the bases called (i.e., when more than 30% of the bases were reported as "N"s) were removed from the dataset and are not further considered here. Sequences with no informative (< e^-5^) matches to any reported sequence were assigned to the "eukaryotic" population, on the grounds that the genomes of bacteria and viruses, which have little or no intergenic material, are more likely to produce at least a partial match on a *tblastx *search. Positions of introns were predicted using GENSCAN [36] and confirmed by alignment and sequence comparison.

Robust regression [37] was employed to estimate a best linear model for the dependence of the observed number of reads on sequence length, and to assign weights measuring each observed point's agreement with the model.

### Identification of mitochondrial sequences

The WGA *A. rara *template was PCR-amplified with specific *A. rara *mitochondrial primers designed using the contig data to obtain longer mitochondrial sequences. The COX1 gene was assumed to occur upstream of COX3 on *A. rara *chromosomes, as is usual in mitochondria. *A. rara *COX1-specific forward primer 5'-GATGGAGGAGTAAATGCTGGTTGAAC-3' and *A. rara *COX3-specific reverse primer 5'-AAACCACCCGAAACGACCAAATG-3' were designed based on contigs 110 and 2876, respectively. The COX1-COX3 PCR were performed on a Techne Genius thermocycler for 40 cycles of 94°C for 30 s, 60°C for 30 s, and 70°C for 2 min. Amplimer sequences were *blastx *searched using the invertebrate mitochondrial genetic code. About 450 bp of an ATP synthase-like gene fragment was identified between the fragments of COX1-like and COX3-like genes in one amplimer sequence [GenBank:HM119599]. Based on this sequence, *A. rara *ATP synthase-specific forward primer 5'-TCTGACGAGGAAGGATGAACATTAGG-3' was designed and paired with the COX3 reverse primer in another PCR amplification under the same cycling conditions. All amplimers were cloned and sequenced as described above.

## Authors' contributions

AH carried out the library construction, performed contig assembly and sequence analysis, and drafted the manuscript. YH designed and carried out the experiments for detection of NUMT and contributed to the manuscript. AAR performed the statistical analysis. SSB collected individuals of *Astrammina rara*, participated in the design of the study, and contributed to the manuscript. All authors read and approved the final manuscript.

## Supplementary Material

Additional File 1 **Identification of probable gene sequence in contigs**. A listing of all eukaryotic contigs which contain identifiable sequence homology to known genes. Click here for file

Additional File 2 **Newbler assembly statistics**. Statistics for the contig assembly performed using the GS FLX de novo assembly tool. Click here for file
