# Supplementary material for: High-throughput sequencing of Astrammina rara: Sampling the giant genome of a giant foraminiferan protist
Source: BMC Genomics. 2011 Mar 31;12:169. doi: 10.1186/1471-2164-12-169 (PMC3079666; doi:10.1186/1471-2164-12-169)
Supplement: Additional File 1 — Identification of probable gene sequence in contigs. A listing of all eukaryotic contigs which contain identifiable sequence homology to known genes. [file 1471-2164-12-169-S1.DOC]

| **Contig** | **Gene function as inferred from BLAST** | **E-value of closest tblastx match**  **(cutoff e -05)** |
| --- | --- | --- |
| 00836 | Myosin | e -95 |
| 02689 | HSP60 | e -81 |
| 00061 | 26S proteasome regulatory particle non-ATPase subunit8 | e -75 |
| 03296 | ABC transporter protein (7 matching domains) | e -66 |
| 02594 | GMP synthetase | e -57 |
| 00014 | Actin 1 | e -57 |
| 03015 | Glutaminyl-tRNA synthetase | e -53 |
| 01403 | *Astrammina* SSU | e -50 (blastn) |
| 01029 | DEAH-box protein | e -48 |
| 00458 | Vacuolar or lysosomal proton-translocating ATPase | e -46 |
| 03080 | Pyrophosphatase or kinase | e -43 |
| 02707 | Ribosomal protein S18 | e -39 |
| 01839 | Histone 2 | e -38 |
| 01104 | Thioredoxin reductase | e -37 |
| 03052 | Aldolase | e -36 |
| 00056 | G-protein coupled 7-transmembrane receptor | e -35 |
| 02865 | Actin 2 | e -34 |
| 03277 | HSP60 | e -34 |
| 01045 | Malate dehydrogenase | e -32 |
| 01161 | Predicted protein of unknown function | e -31 |
| 00068 | Ribosomal protein L21 | e -29 |
| 00070 | Proline dehydrogenase | e -29 |
| 01538 | Class V myosin | e -28 |
| 02338 | HSP70 | e -28 |
| 02652 | Predicted protein of unknown function | e -26 |
| 03201 | SDA1-domain containing protein | e -26 |
| 00033 | DEAD-box or DEAH-box helicase | e -25 |
| 00094 | Predicted protein of unknown function | e -25 |
| 01612 | DEAH-box protein | e -24 |
| 01647 | Myosin | e -24 |
| 00076 | DEAH-box protein | e -23 |
| 03056 | Proton pump | e -23 |
| 00027 | Predicted protein of unknown function | e -22 |
| 02872 | Predicted protein of unknown function | e -21 |
| 00110 | mitochondrial COX1 | e -18 |
| 00551 | Proteasome protein | e -18 |
| 00773 | Predicted protein of unknown function | e -18 |
| 01158 | Beta-tubulin | e -20 |
| 00044 | DEAH-box protein | e -17 |
| 00101 | Predicted protein of unknown function | e -17 |
| 01385 | Class V myosin | e -17 |
| 03159 | Transcription initiator or chromatin remodeling protein | e -17 |
| 01628 | RNA helicase | e -16 |
| 03051 | Tandem array of tRNA genes | e -22 blastn |
| 00048 | DEAH-box protein | e -15 |
| 00100 | Mixed protist ITS regions | e -15 |
| 00875 | Predicted protein of unknown function | e -15 |
| 01003 | Dehydrodolichyl diphosphate synthase | e -15 |
| 01398 | Predicted protein of unknown function | e -15 |
| 01915 | Exportin | e -15 |
| 02030 | Predicted protein of unknown function | e -15 |
| 02441 | Coatomer protein complex subunit | e -15 |
| 03118 | DEAH-box protein | e -15 |
| 00032 | Polyubiquitin or ubiquitin fusion protein | e -14 |
| 00078 | Chromatin regulator | e -14 |
| 02666 | Predicted protein of unknown function | e -14 |
| 03128 | Repeat region | e -14 |
| 00024 | DEAH RNA helicase | e -13 |
| 01098 | Aldolase | e -13 |
| 02033 | Predicted protein of unknown function | e -13 |
| 02206 | 5' - 3' exoribonuclease | e -13 |
| 02240 | Repeat region | e -13 |
| 00997 | Beta tubulins | e -12 |
| 02455 | HSP70s | e -12 |
| 02530 | Predicted protein of unknown function | e -12 |
| 02713 | Repeat region | e -12 |
| 00722 | Coiled-coil protein | e -11 |
| 00906 | Sulfatase modifying factor | e -11 |
| 02452 | Ribosomal protein L27A | e -11 |
| 02509 | Predicted protein of unknown function | e -11 |
| 02599 | Myotubularin protein | e -11 |
| 02843 | Heat shock factor 2 | e -11 |
| 03012 | Repeat region | e -11 |
| 03121 | DNA repair protein | e -11 |
| 00082 | Dynein light chain | e -10 |
| 00339 | Predicted protein of unknown function | e -10 |
| 02590 | Microsatellite sequence | e -10 |
| 02756 | Sulfatase | e -10 |
| 03181 | Histidine-rich protein | e -10 |
| 00396 | Predicted protein of unknown function | e -09 |
| 00456 | Peroxin | e -09 |
| 00468 | Fat-spondin | e -09 |
| 00741 | Exportin/nuclear export factor | e -09 |
| 00938 | Ankyrin domain protein | e -09 |
| 01374 | Predicted protein of unknown function | e -09 |
| 01519 | Repeat region | e -09 |
| 01532 | Predicted protein of unknown function | e -09 |
| 01692 | Repeat region | e -09 |
| 01708 | Repeat region | e -09 |
| 01862 | Predicted protein of unknown function | e -09 |
| 02234 | Repeat region | e -09 |
| 00522 | Predicted protein of unknown function | e -08 |
| 00712 | Translation initiation factor | e -08 |
| 00867 | Kinase | e -08 |
| 00930 | Transportin | e -08 |
| 00993 | Vacuolar ATP synthase | e -08 |
| 01800 | HSP40 | e -08 |
| 02398 | Repeat region | e -08 |
| 02534 | Predicted protein of unknown function | e -08 |
| 02582 | Splicing factor | e -08 |
| 03008 | Repeat region | e -08 |
| 00333 | Predicted protein of unknown function | e -07 |
| 00412 | Repeat region | e -07 |
| 00416 | Very mixed euk hits. Possible tRNAs. | e -07 |
| 00691 | Repeat region | e -07 |
| 00717 | Ankyrin repeat containing protein | e -07 |
| 00739 | Repeat region | e -07 |
| 01124 | Repeat region | e -07 |
| 01154 | Repeat region | e -07 |
| 01303 | Predicted protein of unknown function | e -07 |
| 01389 | Arginine methyltransferase | e -07 |
| 01761 | Predicted protein of unknown function | e -07 |
| 02072 | Repeat region | e -07 |
| 02080 | Repeat region | e -07 |
| 02220 | Predicted protein of unknown function | e -07 |
| 02264 | Predicted protein of unknown function | e -07 |
| 02516 | Receptor-like kinase | e -07 |
| 02554 | Methyltransferase | e -07 |
| 02828 | Transducin | e -07 |
| 03013 | Guanine monophosphate synthetase | e -07 |
| 03267 | Predicted protein of unknown function | e -07 |
| 00013 | Actin | e -06 |
| 00421 | Predicted protein of unknown function | e -06 |
| 00517 | Pol-like protein | e -06 |
| 00523 | Prestin or solute-carrier protein | e -06 |
| 00558 | Myotubalarin-related protein | e -06 |
| 00654 | Repeat region | e -06 |
| 00681 | Predicted protein of unknown function | e -06 |
| 00894 | Repeat region | e -06 |
| 00897 | Repeat region | e -06 |
| 00911 | Polyprotein | e -06 |
| 00925 | Elastin | e -06 |
| 01061 | Repeat region | e -06 |
| 01818 | Microsatellite | e -06 |
| 02031 | Helicase | e -06 |
| 02299 | Repeat region | e -06 |
| 02503 | Repeat region | e -06 |
| 02520 | Repeat region | e -06 |
| 02566 | Repeat region | e -06 |
| 02804 | Mitochondrial COX 1 | e -06 |

Table 1: Contigs with matches < e-5 by BLAST search against eukaryotic sequences.
