# Supplementary material for: High-throughput sequencing of Astrammina rara: Sampling the giant genome of a giant foraminiferan protist
Source: BMC Genomics. 2011 Mar 31;12:169. doi: 10.1186/1471-2164-12-169 (PMC3079666; doi:10.1186/1471-2164-12-169)
Supplement: Additional File 2 — Newbler assembly statistics. Statistics for the contig assembly performed using the GS FLX de novo assembly tool. [file 1471-2164-12-169-S2.DOC]

| Total number of reads: | 234301 |
| --- | --- |
| Number of bases: | 49036585 |
| numberAssembled: | 102022 |
| numberPartial | 12894 |
| numberSingleton | 8049 |
| numberRepeat | 108358 |
| numberOutlier | 2978 |
| Largest contig | 13,634 |
| Q40PlusBases | 857012, 89.84% |
| Q39MinusBases | 96950, 10.16% |

Table 2: Newbler assembly statistics.
